# BATHING, MAKE-UP, AND SUNSCREEN: WHICH PRODUCTS DO CHILDREN USE?

**DOI:** 10.1590/1984-0462/2020/38/2018319

**Published:** 2020-05-08

**Authors:** Thayane Guimarães de Melo, Mayara Schulze Cosechen Rosvailer, Vânia Oliveira de Carvalho

**Affiliations:** a Universidade Federal do Paraná, Curitiba, PR, Brazil.

**Keywords:** Cosmetics, Personal hygiene products, Children, Adolescent, Cosméticos, Produtos para higiene pessoal, Crianças, Adolescente

## Abstract

**Objective:**

To evaluate the hygiene practices and frequency of use of personal hygiene products, cosmetics, and sunscreen among children and adolescents.

**Methods:**

Cross-sectional study with interviews about skincare conducted with caregivers through closed-ended questions. We included patients up to 14 years of age waiting for consultation in pediatric outpatient clinics of a tertiary hospital. We performed a descriptive statistical analysis and applied the Kruskal-Wallis test and Fisher’s exact test to compare the practices according to maternal schooling.

**Results:**

We conducted 276 interviews. The median age of the participants was age four, and 150 (54.3%) were males. A total of 143 (51.8%) participants bathed once a day and 128 (46.3%) bathed two or more times a day, lasting up to ten minutes in 132 (47.8%) cases. Adult soap was used by 103 (37.3%) children and bar soap by 220 (79.7%). Fifty-three (19.2%) participants used sunscreen daily. Perfume was used by 182 (65.9%) children, hair gel by 98 (35.5%), nail polish by 62 (22.4%), and some type of make-up by 71 (25.7%) - eyeshadow by 30 (10.8%), lipstick by 52 (18.8%), face powder and mascara by 13 (4.7%). Make-up use started at a median age of 4 years. Henna tattoo was done in eight children.

**Conclusions:**

The children studied used unsuitable products for their skin, such as those intended for adults, used sunscreen inadequately, and started wearing make-up early, evidencing the need for medical orientation.

## RESUMO

**Objetivo:**

Avaliar os hábitos de higiene e a frequência do uso de produtos de higiene pessoal, cosméticos e protetor solar nas crianças e adolescentes.

**Métodos:**

Estudo transversal com entrevistas para cuidadores sobre cuidados com a pele, por meio de perguntas fechadas. Incluídos pacientes de até 14 anos que consultavam nos ambulatórios pediátricos de um hospital terciário. Realizada estatística descritiva e aplicados os testes de Kruskal-Wallis e exato de Fisher para comparar os hábitos conforme a escolaridade materna.

**Resultados:**

Foram realizadas 276 entrevistas. A mediana de idade foi de 4 anos, sendo 150 (54,3%) crianças do sexo masculino. O número de banhos por dia foi de um em 143 (51,8%) casos e dois ou mais por parte de 128 (46,3%) indivíduos, com duração de até dez minutos em 132 (47,8%) dos participantes. O sabonete destinado a adultos era utilizado por 103 (37,3%) crianças e o sabonete em barra por 220 (79,7%) delas. Protetor solar era utilizado diariamente por 53 (19,2%) participantes. Perfume foi utilizado por 182 (65,9%) integrantes da amostra, gel de cabelo por 98 (35,5%), esmalte por 62 (22,4%) e algum tipo de maquiagem por 71 (25,7%) - sombra em 30 (10,8%), batom em 52 (18,8%), pó facial e rímel em 13 (4,7%). A mediana de idade de início do uso de maquiagem foi de 4 anos. Tatuagem de hena foi realizada em oito crianças.

**Conclusões:**

As crianças estudadas utilizavam produtos inadequados para a sua pele, como os destinados à pele do adulto, e faziam uso incorreto do protetor solar e uso precoce de maquiagem, mostrando a importância da orientação médica.

## INTRODUCTION

Skin is the barrier between the body and the external environment. The epidermis is the outermost layer of skin, and the stratum corneum (SC) plays the role of barrier. This barrier is both physical, preventing the invasion of pathogens, and chemical, due to its acidic pH, in addition to acting in the maintenance of skin hydration.^1^ Preserving the characteristics of the skin barrier is essential,^2^ and, therefore, hygiene products, cosmetics, and photoprotectors intended for children should be formulated accordingly.^1^

Skin pH is necessary to maintain the homeostasis of the skin barrier, for the integrity and cohesion of the SC, and also in antimicrobial defense. The normal skin pH is acidic, ranging from 4 to 6, known as the acid mantle, and protects the body against the proliferation of microorganisms. The enzymes involved in the synthesis and maintenance of the skin barrier are influenced by pH changes caused, for example, by bar soaps, which are alkaline.^3^ The permeability and the pH of the SC are interdependent, and their changes can facilitate the development of skin diseases, such as atopic dermatitis and contact dermatitis.^1^ ^,^ ^4^

Proper skin hygiene in children is fundamental to eliminate potentially irritating substances,^1^ but the products used in this population should be mild, without fragrance, and with slightly acidic pH.^5^ ^,^ ^6^ Due to the lower concentration of sebum in the skin of children until the start of adolescence, their hygiene products do not need to have powerful detergent action.^7^

The frequency of bathings varies in each country, influenced by individual, cultural, and social factors,^8^ but the different processes adopted can determine the maintenance of or modification in the skin barrier. Bathings should be short and use warm water to minimize transepidermal water loss.^7^ ^,^ ^9^ Hygiene items must be water-based, of low-fixation, and not present oral toxicity to be classified as children’s products.^10^ Using hygiene products and cosmetics for children is crucial since their skin will start resembling that of an adult as an effective barrier after the age of three to four years, and in producing sebum after the age of ten to 12 years.^1^ ^,^ ^4^

Moreover, estimates indicate that up to 80% of the cumulative sun exposure of a person occurs during childhood; therefore, photoprotection is important in this life stage.^11^ Sunscreen prevents skin cancer,^11^ ^,^ ^12^ however, the proportion of the pediatric population that uses this product daily is far from adequate.^13^ ^,^ ^14^ ^,^ ^15^ ^,^ ^16^

In this context, this study aimed to evaluate the hygiene practices and frequency of use of personal hygiene products, cosmetics, and sunscreen among the pediatric population.

## METHOD

We conducted a cross-sectional study, which included patients up to 14 years of age who were waiting for consultation in pediatric outpatient clinics (allergology, cardiology, dermatology, genetics, gastroenterology, general pediatrics, and pediatric surgery) of a tertiary hospital, from March 4, 2015 to March 30, 2016, after approval by the Human Research Ethics Committee of the institution.

The participants’ caregivers were interviewed based on a structured questionnaire on daily hygiene care and the use of sunscreen and cosmetics, such as make-up. We analyzed the variables: maternal age and schooling, patient’s age and gender, the number of weekly bathings, type of bathing (shower, bath, or both), bathing duration, use of soap and soap type, use of shampoo and shampoo type, use of moisturizer, sunscreen, hair gel, and other cosmetics. Regarding make-up, the questions concerned the use of lipstick, nail polish, eyeshadow, eye pencil, liquid eyeliner, mascara, foundation/face powder, and blush. We also inquired about the starting age of and what motivated make-up use, as well as the use of henna tattoo and hair dye.

Researchers administered the questionnaires in the waiting room of outpatient clinics of the hospital, in the following weekdays: Mondays and Fridays in the afternoon; and Wednesdays during the morning shift. We included all children waiting for consultation during this period, regardless of their diagnosis or specialization of the outpatient clinic treating them, who agreed to participate in the study and whose parent/guardian signed the informed consent form. The study had no exclusion criteria.

The statistical analysis was performed in the software R, version 3.3.1.; data were expressed as frequencies, mean, and median; and we applied the Kruskal-Wallis test and Fisher’s exact test to compare the use of sunscreen and the starting age of make-up use with maternal schooling, considering a 5% significance level.

## RESULTS

A total of 276 caregivers of children waiting for consultation were interviewed. In 179 (64.8%) cases, the caregiver was the child’s mother. Regarding maternal schooling, 100 (36.2%) mothers had elementary school education; 134 (48.5%), high school education; and 35 (12.6%), higher education. In seven (2.5%) cases, the caregiver did not know the maternal schooling.

Among the children participating, the median age was 4 years (10 days to 14 years), and 150 (54.3%) were males. The distribution by age group was two newborns (0.7%), 73 (26.6%) infants, 113 (40.9%) preschoolers, 60 (21.7%) schoolchildren, and 28 (10.1%) adolescents.

As to the children’s hygiene, 143 (51.8%) participants bathed once a day, 194 (70.2%) bathed in the shower, and in 144 (52.1%) cases, the bathings lasted more than 10 minutes. The water temperature was considered warm by 222 (80.4%) participants. Table 1 presents the remaining data.

**Table 1:** Distribution of hygiene practices and use of cosmetics among the participants.

Hygiene practices	n	%	Median age
Bathing frequency			
Less than once a day	5	1.8	1 month
Once a day	143	51.8	5 years
Two or more times a day	128	46.4	3 years
Bathing type			
Shower	194	70.3	6 years
Bath	73	26.4	1 year
Bath+shower	9	3.3	1 year
Bathing duration			
Up to 5 minutes	40	14.5	7 years
5 to 10 minutes	92	33.3	7 years
More than 10 minutes	144	52.2	8 years
Water temperature			
Hot	49	17.7	5 years
Warm	222	80.4	4 years
Cold	5	1.8	3 years

Adult soap was used by 103 (37.3%) children. In regard to the form of soap, 220 (79.7%) participants used bar soap. Children’s shampoo was used by 182 (65.9%) individuals (Table 2) and sponge by 126 (45.6%).

**Table 2:** Distribution of the use of cosmetics among the participants.

Use of cosmetics	n	%	Median age
Soap form			
Bar	220	79.7	5 years
Liquid	51	18.5	1 year
Bar+liquid	4	1.4	4 years
Does not use	1	0.4	2 years
Soap type			
Children	156	56.5	2 years
Adult	103	37.3	7 years
Medical prescription	16	5.8	4 years
Does not use	1	0.4	2 years
Shampoo type			
Children	182	65.9	3 years
Adult	75	27.2	9 years
Medical prescription	5	1.8	3 years
Does not use	14	5.1	1 year
Type of make-up used			
Lipstick	52	18.8	7 years
Eyeshadow	30	10.9	7 years
Face powder/blush	13	4.7	9 years
Mascara	13	4.7	9 years
Foundation	8	2.9	7 years
Eyeliner	7	2.5	6 years

Out of all participants, 112 (40.5%) did not use emollient after bathing, and, among those who did, 107 (38.7%) used it daily and 57 (20.6%), sporadically.

**Figure 1:**
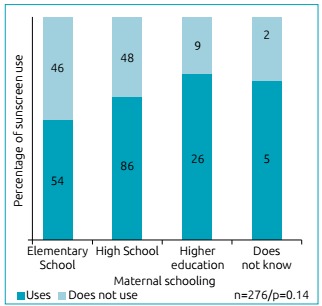
Distribution of sunscreen use according to maternal schooling.

Regarding sunscreen, 106 (38.6%) participants never use the product. Fifty-three (19.2%) children applied it daily; 105 (38.4%), only in case of sun exposure; and 12 (4.35%), occasionally. Maternal schooling was not associated with the use of sunscreen (Figure 1, p=0.14).

The number of children who used hair gel was 98 (35.5%); perfume, 182 (65.9%); deodorant, 37 (13.4%); and nail polish, 62 (22.4%).

Seventy-one (25.7%) participants wore some type of make-up, such as eyeshadow, lipstick, foundation, face powder or blush, and mascara. Among them, two (2.8%) were infants; 32 (45.0%), preschoolers; 28 (39.4%), schoolchildren; and nine (12.7%), adolescents. Table 2 shows the frequency of each product. Make-up use started at the median age of 4 years (1 to 11 years). The starting age of make-up use showed no statistically significant relationship with maternal schooling. In mothers with elementary school, high school, and higher education, the starting age was, respectively, 4.8, 4.0, and 4.2 years (p=0.66). The reason for the use of cosmetics was the child’s request in 53 (74.6%) cases, the desire of the parents in eight (11.2%), and the influence of others in 10 (14.2%).

Henna tattoo was done in eight (2.8%) participants, with ages ranging from 4 to 12 years, and six (2.1%) children, aged 5 to 12 years, used hair dye.

## DISCUSSION

The data presented revealed that the skin hygiene in children is not adequate, with bathing frequency and duration higher than the recommended, use of adult products, and application of sunscreen only in case of sun exposure.

Children’s skin is 30% thinner than that of adults until the age of 3 years, in addition to losing more liquid and having greater absorption capacity.^1^ Therefore, children should only use products intended for them.^5^ ^,^ ^9^ ^,^ ^17^ Bathings with liquid soaps that do not alter the pH of the skin surface or cause irritation have proven to be more efficient than those with only water.^18^ Syndets (soaps with synthetic surfactants) are based on neutral or acid synthetic detergents, eliminating fewer lipids and minimizing transepidermal water loss when compared with other soaps.^7^ Hygiene agents based on soaps are alkaline and more likely to cause skin irritation.^3^ The present study indicated that 37.3% of the children used adult soap, which can lead to changes in the homeostasis of the skin barrier. The use of suitable soaps that do not alter the acidic skin pH must be part of the instructions for child hygiene.

The ideal bathing duration, especially among children with dry skin, should not exceed 10 minutes,^19^ which was identified in 47.8% of the children in this study. Long bathings increase transepidermal water loss.^7^ Thus, 52.1% of the children studied might have the characteristics of the skin barrier damaged due to longer bathings. Bathings with adequate temperature (37.0-37.5°C), found in 80.4% of the population studied, preserve the thermal stability and do not increase transepidermal water loss^7^.

The use of emollients in patients with xerotic skin improves the efficiency of the skin barrier.^20^ The benefit of using emollients routinely in children without xeroderma still requires further studies.^1^ In the population assessed, 59.4% used emollients. Among these individuals, 38.7% used it daily, which is more effective in protecting and maintaining the skin barrier than the sporadic use^5^ declared by the other participants.

Proper photoprotection for all age groups is an essential public health measure to prevent skin neoplasms^11^ ^,^ ^12^ as well as photoaging.^11^ In the sample investigated, 61.9% of the children used sunscreen, but only 19.2% on a daily basis. In 2012, Dupont and Pereira conducted a study in the city of Carlos Barbosa, in Southern Brazil, and revealed that 8.1% of the pediatric population studied used sunscreen daily.^13^ An investigation carried out with preschoolers from public and private schools of a city in Santa Catarina, Brazil, found that 4.4% of the sample applied sunscreen daily.^16^ In Porto Alegre, Brazil, caregivers were questioned regarding the application of sunscreen in children, and 18.6% of them declared using it daily. These data indicate the need for educational campaigns for photoprotection targeted at children and adolescents^21^ and their parents/guardians,^22^ given that sun exposure at early life stages influences the onset of skin neoplasms, and sun protection practices acquired in childhood and adolescence can change future behaviors.^11^

We found increasingly early use of make-up, such as lipstick, lip gloss, and eyeshadow, in the pediatric population.^9^ The children participating in this study started wearing make-up early, regardless of maternal schooling, as reported by Biesterbos in 2013.^23^ A quarter of the sample investigated (25.7%) used some type of make-up, starting at a median age of 4 years. Few studies in the literature have evaluated the frequency of use of cosmetics among the pediatric population. In 2015, research conducted in France underlined that the use of make-up in girls aged 4 to 14 years ranged between 11 and 19%.^24^ In California, 60% of children aged 2 to 5 years wore lipstick.^25^ Children might be more susceptible to the effects of exposure to chemical agents because they have an immature immune system and a greater proportional body surface area.^17^ The impact of the frequent use of make-up may not be immediately visible, emerging only after years of exposure.^12^ The prevalence of contact dermatitis in the pediatric age group has increased progressively, due to both the improvement in diagnostic accuracy and the higher incidence of this condition, caused by greater exposure to potentially allergenic products.^26^ ^,^ ^27^ When the cause for contact dermatitis cannot be determined, morbidity might be significant because of the chronicity and recurrence of the lesions.^26^ Thus, patients and their caregivers should be aware of the possible risks involving the use of make-up in childhood, such as contact dermatitis^9^ ^,^ ^28^ and exacerbation of atopic dermatitis.^28^ ^,^ ^29^ We emphasize that skin products should be used with caution in children to avoid potentially allergenic substances.^30^

A limitation of this study is the sample consisting of children treated in a tertiary hospital. Therefore, they might have received instructions regarding hygiene and photoprotection practices, which could overestimate the use of sunscreen and the number of children with appropriate hygiene practices. The results and discussion, however, showed that the pediatric population studied still presented inadequate hygiene practices and use of sunscreen, used products intended for adult skin, and wore make-up early.

Health professionals should be informed about this reality in order to instruct parents and guardians, and awareness campaigns on the theme should be developed and implemented.
